# PD38 Ensuring Study Validity To Inform Health Technology Assessments Globally

**DOI:** 10.1017/S0266462324003015

**Published:** 2025-01-07

**Authors:** (Emily) Beth Devine, Penny Whiting, Sue Mallett, Robert Wolff, Jelena Savovic

## Abstract

**Introduction:**

Assurance that supporting evidence is based on valid and unbiased assessments, evaluated using rigorously developed risk of bias (validity assessment) tools, is fundamental to good decision-making. Among those available, selecting and correctly using the best tool that is fit-for-purpose is challenging. Collaboration across the global evidence synthesis and health technology assessment (HTA) communities promotes best practices and harmonizes tool use across jurisdictions.

**Methods:**

We have established the LATITUDES Network (https://www.latitudes-network.org/), a publicly available website library of validity assessment tools and resources to guide decision-makers in selecting and applying tools appropriate for particular contexts, including informing HTA reimbursement decisions, clinical guideline development, and stand-alone evidence synthesis projects. The internationally representative leadership team comprises five evidence synthesis experts who have been supported by a competitively awarded academic innovations grant. The 23-member advisory panel representing five continents provided expertise to finalizing criteria for tool inclusion, identifying key tools, and suggesting inclusion of additional tools. Formal launch took place at the 2023 Cochrane Colloquium.

**Results:**

Officially launched in September 2023, the LATITUDES Network indexes validity assessment tools developed for healthcare studies in an online library. To date, 10 key tools are featured to help reviewers identify the optimal tool for their use. Nineteen additional tools have met all screening criteria and are also recommended. Information characterizing each tool (e.g., citation and training materials) is provided. Seven tools are currently under development. A mechanism for users to suggest new tools is provided. Additional tools and information on toolkits and online training materials, as well as links to courses and events, will be added over time.

**Conclusions:**

LATITUDES aims to be the primary resource that provides key information to reviewers conducting validity assessments for evidence synthesis, clinical guideline development, and HTA decision-making. It is intended to increase the robustness of evidence synthesis by improving the process of validity assessment, helping scientists use tools more effectively and efficiently, promoting best practices, and harmonizing validity assessment across the globe.

